# Efficacy of irrigation activation methods in removal of bioceramic-based sealer in retreatment

**DOI:** 10.1007/s10266-025-01054-z

**Published:** 2025-02-08

**Authors:** Büşra Nur Cıkrık, Selen İnce Yusufoğlu

**Affiliations:** 1 Private Turadent Oral and Health Clinic, Istanbul, Türkiye; 2 Department of Endodontics, Ankara Yıldırım Beyazıt University Faculty of Dentistry, Ankara, Türkiye

**Keywords:** MTA Fillapex, Bioserra, EDDY, Retreatment, Activation methods, Bioceramic-based sealers, SEM

## Abstract

In cases where initial root canal therapy is unsuccessful, retreatment is the first treatment choice. However, when bioceramic-based sealers have been used in the initial treatment, their complete removal can be challenging. The objective of this study was to evaluate the efficacy of three final irrigation activation methods-conventional needle irrigation, passive ultrasonic activation, and EDDY activation—using a scanning electronic microscopy (SEM) to evaluate the removal of bioceramic-based sealers after retreatment. A total of 108 single-rooted teeth were prepared using ProTaper Next rotary files. The samples were obturated with either MTAFillapex or BioSerra (*n* = 54) and stored for 2 weeks post-obturation. After the retreatment procedures, the teeth were divided into six groups (*n* = 18 per group) based on final irrigation activation method used. Group1:MTAFillapex-CNI, Group2:BioSerra-CNI, Group3:MTAFillapex-EDDY, Group4:Bioserra-EDDY, Group5:MTAFillapex-UltraX, Group6:Bioserra-UltraX. The roots were sectioned longitudinally, and the coronal, middle and apical parts were investigated under a SEM. The data were then statistically analysed. The EDDY activation technique proved significantly more effective in removing MTA Fillapex across all sections of the root canal (*p* < 0.001). In contrast, Ultra-X was more effective in removing Bioserra in the middle and apical sections (*p* = 0.003, *p* < 0.001). These findings suggest that activation procedures may be preferable for removing bioceramic-based root canal sealer.

## Introduction

The purpose of the retreatment is to ensure adequate cleaning, reshaping and filling of the root canal system by removing previous root canal filling materials, residues and microorganisms [1]. The thorough removal of the original obturation is critical to reach contaminated regions, including dentinal tubules, isthmuses, and lateral canals, thereby facilitating successful disinfection [2]. If the original root canal filling is not entirely eliminated, it may form a barrier between the dentin surface and the new obturation material, hinder the proper setting of the root canal sealer, and increase the risk of coronal or apical microleakage [3]. The residual root canal filling material may persist in the root canal system after retreatment [4]. Consequently, it is advised to use additional technique to remove any remaining filling material [4].

Bioceramic-based root canal sealers are highly hydrophilic and can chemically bond to dentin [5]. Studies show that these sealers, widely used in endodontics, promote dentin mineralization, exhibit acceptable cytotoxicity levels, possess antibacterial properties, and achieve good penetration into dentinal tubules [5, 6]. The use of bioceramic-based root canal sealer has won popularity in endodontics [5, 6]. *Bioserra* (Metabiomed, Osongsaengmyeong 1-ro, Korea) is a bioceramic-based sealer with a white, fluid paste consistency, designed for easy application using a disposable tip attached to a pre-filled syringe. Its composition includes calcium silicate, zirconium oxide, and aluminum as filler materials [7]. Another notable bioceramic-based selaer is *MTA Fillapex* (Angelus, Londrina-PR Brazil) which consist of bismuth trioxide, natural resin, diluted resin, salicylate resin, and nanoparticle silica [8]. It demonstrates low expansion during hardening and limited solubility when in contact with tissue fluids. Additionally, since it does not contain eugenol, it avoids negatively affecting the setting of resin-based cements [9].

Although it is easy to remove gutta-percha, which is used as the main root canal obturation material during retreatment procedure [10], there are concerns about the use of bioceramic-based sealers due to the limited evidence available regarding their removability [11]. Bioceramic-based root canal sealers have been observed to be difficult to remove from the root canal system because of their very rigid structure once set and their interaction with dental tissues after an unsuccessful root canal treatment [12, 13]. Some experts and academics have expressed concerns about the retrievability of bioceramic filling materials during endodontic retreatment [14, 15]. Obstacles encountered during re-treatment of bioceramic-based obturations include difficulty in determining the working length, extended procedure time, and incomplete cleaning of the canal walls [11, 16].

Numerous methods, including hand files and rotary or reciprocating equipment, have been used to remove root canal fillings [1]. However, studies indicate that none of these techniques can fully eliminate root filling materials, particularly in complex anatomical configurations such as oval-shaped, curved canals, and isthmuses. Modern irrigation activation techniques, including sonic, ultrasonic, and laser methods, have been developed to enhance cleaning. Additional irrigation activation during retreatment has been reported to increase removal of root canal filling material by 1.5–2.1 times [17].

Ultra X (Eighteeth, Changzhou, Chinese), is a wireless device operating at 45 kHz ultrasonic frequency. It utilizes acoustic microflow, agitation and cavitation principles to access areas within the complex root canal system that conventional instruments can not reach [18]. Ultrasonic energy generates acoustic waves and cavitations within the irrigation solution, enhancing its contact with previously inaccessible areas and surfaces. This process helps eliminate the smear layer and residual filling materials from the root canal system [18, 19]. Since ultrasonic metal tips can create a smear layer on dentin, polyamide structured sonic activation systems are used to enhance agitation. EDDY (VDW, Münih, Almanya), is a sonic device powered by an air scaler, operating at approximately 6000 Hz. Due to the EDDY’s disposable polyamid tip, root canal dentin preperation is avoided. The device uses high-frequency vibration to transfer a high amplitude oscillattory movement to the polyamide tip. This enables irrigation activation through three-dimensional movement, which generates cavitation and sonic flow within the irrigation solution [19]. The optimal removal of root canal filling materials during retreatment procedures depends on selecting the appropriate treatment approach for each situation.

In the light of this knowledge, the objective of the study was to evaluate the efficacy of conventional needle irrigation (CNI), Ultra X and EDDY final irrigation activation techniques in the retreatment of teeth obturated with Bioserra and MTA Fillapex root canal sealers, using a scanning electron microscopy (SEM). The null hypothesis stated that there would be no statistically significant variation in the efficay of irrigation activation methods for teeth filled with bioceramic root canal sealers during the retreatment process.

## Materials and methods

### Sample selection

The number of teeth included in the study was determined based on a power analysis conducted using G*Power (G* Power 3.1.7 for Windows, Dusseldorf, Germany) (Effect size: 1.368; Alfa: 0.05; Power: 0.95). The number of samples was 108 (*n* = 18) and it was estimated with a test power of 95%, effect size 1.368 and 5% significance level.

Following approval the ethic committee’s (2023–137), one hundred and eight single-rooted teeth that had been extracted due to orthodontic or periodontal problems included the study. Teeth that were larger than #15 K-file in a diameter at the apical size were not included. After microscopic and radiographic examination, roots devoid of calcification, resorption, or fracture formation were included. All samples were kept in sterile saline solution for 7 days until experiments.

### Sample preparation

A diamond disk (Superapid, Horico, Berlin, Almanya) was used to remove the crown of the teeth. Using a 15-K file (Dentsply, Sirona, Baillagues, Switzerland) the lengths of the root canals were measured up to the point of the instrument was visible from the apical foramen. All teeth had their root canal lengths corrected to 13 ± 1 mm. ProTaper Next (Dentsply Sirona, Ballaigues, Switzerland) X1 (17.04) and X2 (25.06) files were used to instrument the root canals, and two mL of 2.5% NaOCl (Werax, Izmir, Turkey) solutions were used to irrigate them. Torque controlled AI (Woodpecker, Guilin, China) endodontic motor was used for root canal preparation. As per the guideliness provided by the manufacturer all root canal files were used at 300 rpm and 2 Ncm torque. 2 ml of 2.5% NaOCl (Werax, Izmir, Turkey) were applied to the root canal during instrumentation. Following the preparation process, after drying with paper points, the root canals were irrigated with 2 mL of distilled water, 2 mL of 2.5% NaOCl, and 2 mL of EDTA.

### Sample obturation

The prepared roots then obturated using either Bioserra or MTA Fillapex root canal sealers along with ProTaperNext X2 gutta-percha cone (DentsplySirona, Ballaigues, Switzerland).

The excess gutta-percha above the root canal orifice was removed using a heated hand tool. After obturation procedures, the canal orifices were sealed with a temporary filling substance (Cavit-G; 3 M Espe, Seefeld, Germany). To enable the sealer to completely set, the teeth were then kept for 2 weeks at 37 °C and 100% humidity.

### Sample retreatment

**Fig. 1:**
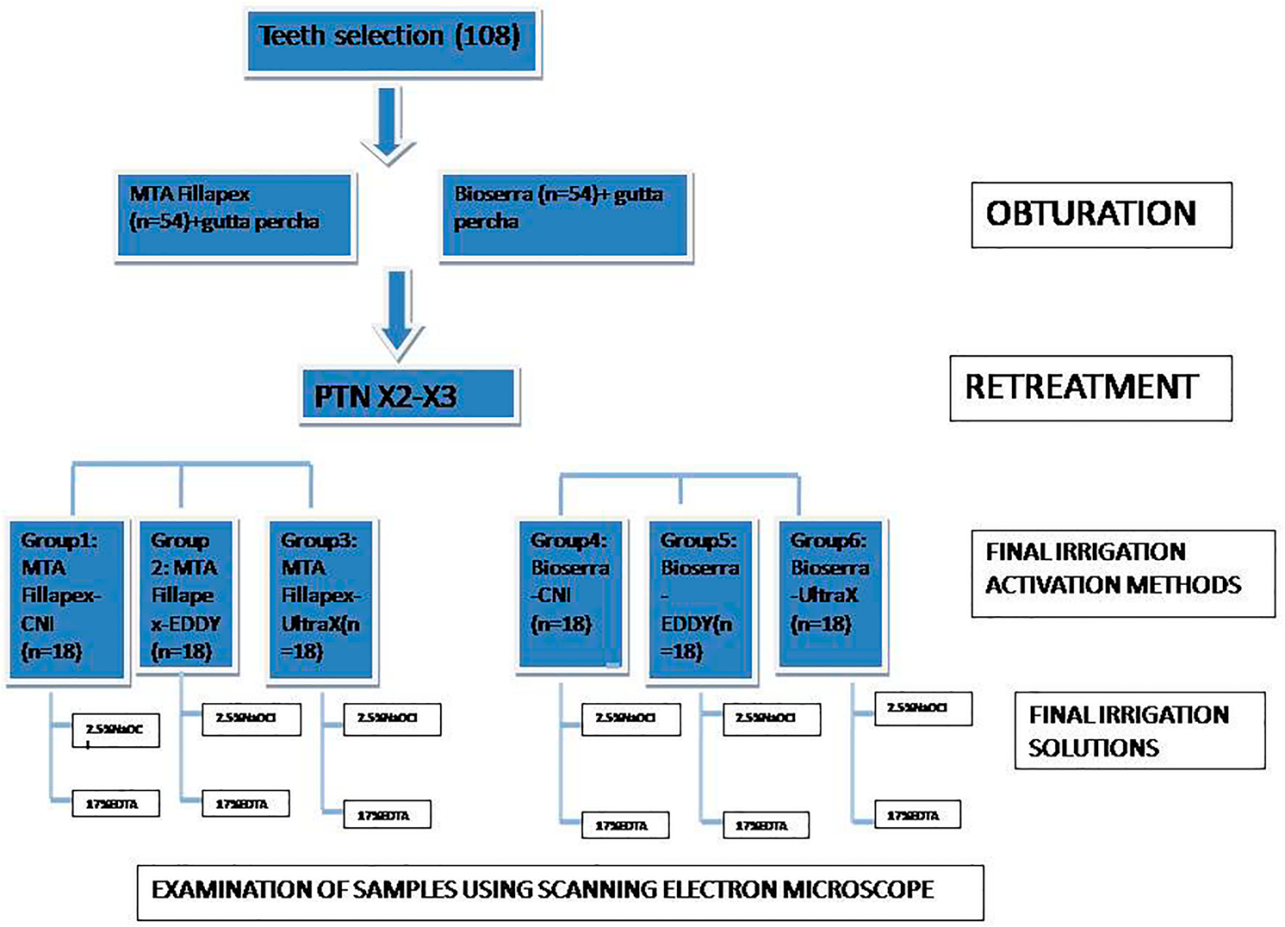
Flowchart illustrating the methodology of the study

Rotary instrumentation was performed using the X2 (25.06) and X3 (30.07) files from the ProTaper Next rotary file system, along with an AI-controlled endodontic motor (500 rpm, 3 N·cm). To retrieve the remaining filling material, the samples were divided into three equal groups based on the additional irrigation activation methods employed: EDDY, Ultra-X, and CNI (Fig. 1).

Group 1–4 (MTA Fillapex-CNI, Bioserra-CNI): The samples in this group did not receive irrigant activation and served as the control group. Following the initial mechanical retreatment stage, the samples were irrigated with a 27-G CNI placed 1 mm short of the WL. The root canals were irrigated with 2 mL of 2.5% NaOCl, over 30 s, with the needle moving in a slow-up-and- down motion. Three times, this procedure was carried out. The same irrigation protocol was applied for 17% EDTA. For the final irrigation, 5 mL of sterile saline was used.

Group 2–5 (MTA Fillapex-EDDY, Bioserra-EDDY): The root canals were irrigated with 2.5 mL of 2.5% NaOCl, using a 27-G needle placed 1 mm short of the WL for 30 s without activation. The irrigation protocol was then modified to accommodate the EDDY TA-200 (Micron, Tokyo, Japan), a 28 mm long polyamide tip with a size of 25.04. The irrigant was activated for 30 s by moving the tip up and down with a 5 mm amplitude at a frequency of 6000 Hz, the highest speed setting. Three times, this procedure was carried out. The same irrigation protocol was applied for 17% EDTA. Finally, 5 mL of sterile saline was used for the final irrigation.

Group 3–6 (MTA Fillapex-UltraX, Bioserra-UltraX): The root canals were irrigated with 2.5 mL of 2.5% NaOCl using a 27-G needle placed 1 mm short of the WL for 30 s without activation. The irrigant was then activated using ultrasonic frequency of 45 kHz, set to the highest power, for 30 s with a size 20/02 silver ultrasonic tip attached to the wireless ultrasonic unit. This process was repeated two more times (30 s × 2). The ultrasonic tip was positioned 2 mm short of the WL. The same irrigation protocol was applied for 17% EDTA. Finally, 5 mL of sterile saline was used for the final irrigation.

### Scanning electron microscopy (SEM) analysis

The samples were grooved longitudinally without penetrating the canal entirely, using a diamond disc mounted on a low speed handpiece and micromotor. The roots were meticulously split into two halves using a chisel. After drying, a sputter-coating device was used to apply a thin layer of gold to the samples, which were then mounted on copper stubs. The optimal half of each root was selected for the SEM examination of the coronal, middle and apical thirds at 2000 × magnification with a 10 kV.

Using the Adobe Photoshop CS4, the SEM images were split into 100 frames for scoring, and the areas containing residual filling materials were quentified. Two endodontists independently and blindly evaluated the SEM images based on the criteria established by Drukteins and Balciunine (2006) [20]:

**Score 1:** 25% of the canal wall is covered by little to no debris.

**Score 2:** Between 25 and 50% of the canal wall is covered in little to moderate debris.

**Score 3:** Between 50 and 75% of the canal wall is covered in moderate to heavy debris.

**Score 4:** Over 75% of the canal wall is covered in large amounts of aggregated or scattered debris.

**Score 5:** Whole debris layer covering the canal wall.

### Statistical analysis

The data were analysed with IBM SPSS v25 (IBM Corporation, Armonk, NY, USA). Data normality was evaluated using the Shapiro–Wilk test. The Kruskal–Wallis and Mann–Whitney U tests were utilized to compare the groups. The Friedman test was performed to determine whether there were significant differences in localization scores when the root canal sealer and activation techniques were kept constant. The Dunn–Bonferroni multiple comparison test was conducted to identify specific differences when the results of the Kruskal–Wallis or Friedman tests were found to be significant. All statistical tests have a significance threshold of *p* < 0.05. Type I errors in multiple comparisons were accounted for using the Bonferroni correction.

## Results

**Fig. 2:**
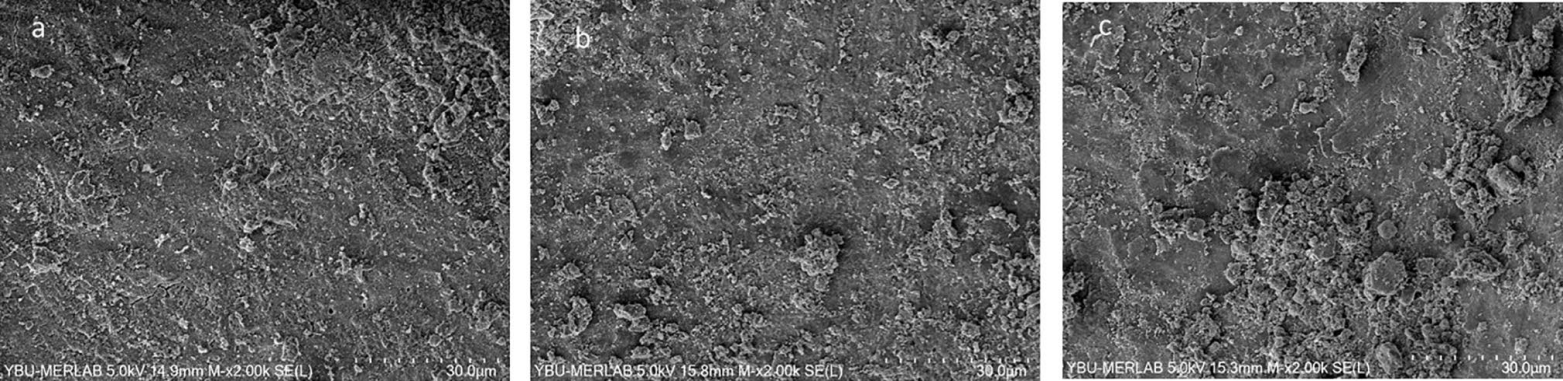
Representative scanning electron microscope images (× 2000) of the **a** coronal third, **b** middle third, **c** apical third after MTAFillapex group with CNI

**Fig. 3:**
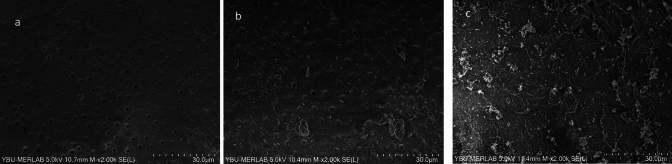
Representative scanning electron microscope images (× 2000) of the **a** coronal third, **b** middle third, **c** apical third after MTAFillapex group with EDDY

**Fig. 4:**
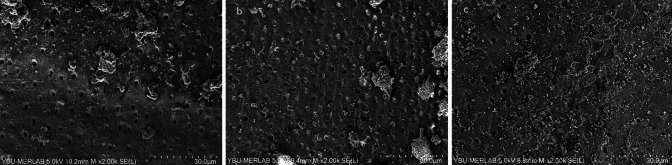
Representative scanning electron microscope images (× 2000) of the **a** coronal third, **b** middle third, **c** apical third after MTAFillapex group with UltraX

**Fig. 5:**
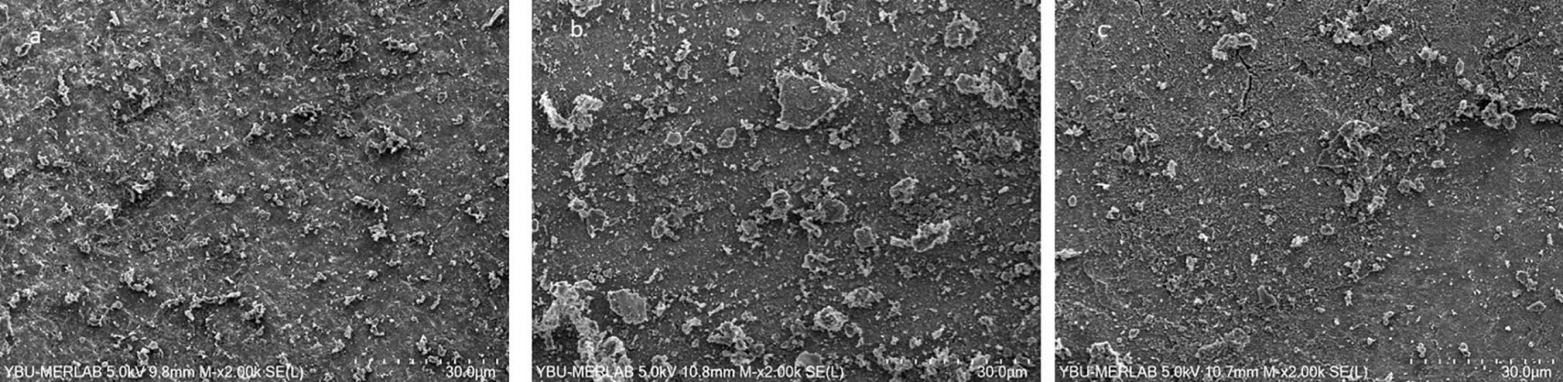
Representative scanning electron microscope images (× 2000) of the **a** coronal third, **b** middle third, **c** apical third after Bioserra group with CNI

**Fig. 6:**
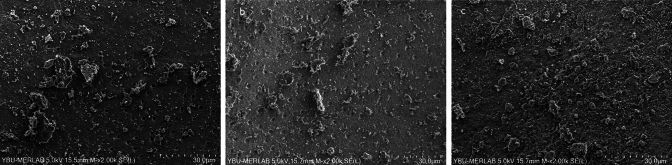
Representative scanning electron microscope images (× 2000) of the **a** coronal third, **b** middle third, **c** apical third after Bioserra group with EDDY

**Fig. 7:**
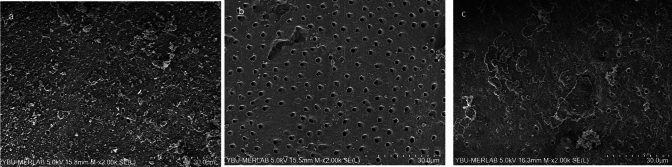
Representative scanning electron microscope images (× 2000) of the **a** coronal third, **b** middle third, **c** apical third after Bioserra group with UltraX

In none of the groups was the root filling material entirely eliminated. Representative SEM microphotographs from each third of the canal wall samples were taken (Figs. 2–7). The median scores at each third of the root canal were recorded and compared for all experimental groups (CNI, Ultra X, EDDY) (Table 1).

**Table 1:** Multiple comparisons between activation methods when location and root canal sealer are kept constant Dunn-Bonferroni test

	EDDY*vs* UltraX			EDDY*vs*CNI			UltraX *vs* CNI		
	EDDY Median(min–max)	UltraX Median (min–max)		EDDY Median (min–max)	CNI Median (min–max)		UltraX Median (min–max)	CNI Median (min–max)	
**Coronal**									
MTA Fillapex	3 (2–5)	4 (3–4)	*p* = 0.192	3 (2–5)	4 (3–5)	***p* < 0.001**	4 (3–4)	4 (3–5)	*p* = 0.108
BioSerra	4 (2–4)	3 (2–4)	*p* = 0.732	4 (2–4)	4 (3–5)	*p* = 0.132	3 (2–4)	4 (3–5)	***p* = 0.004**
**Middle**									
MTA Fillapex	3 (2–5)	4 (3–5)	*p* = 0.595	3 (2–5)	4 (3–5)	***p* < 0.001**	4 (3–5)	4 (3–5)	*p* = 0.024
BioSerra	4 (2–5)	2 (1–4)	***p* = 0.003**	4 (2–5)	4 (3–5)	*p* = 0.541	2 (1–4)	4 (3–5)	***p* < 0.001**
**Apical**									
MTA Fillapex	4 (2–5)	5 (4–5)	*p* = 0.011	4 (2–5)	5 (3–5)	***p* = 0.004**	5 (4–5)	5 (3–5)	*p* > 0.999
BioSerra	4 (2–5)	3 (2–4)	***p* < 0.001**	4 (2–5)	4 (2–5)	*p* > 0.999	3 (2–4)	4 (2–5)	***p* < 0.001**

The bold characters indicate that statistical significance

According to Bonferroni correction, results were considered statistically significant for *p* < 0.0083

In samples where MTA Fillapex root canal sealer was used, EDDY demonstrated significantly better performance than CNI in the coronal, middle, and apical thirds (*p* < 0.001, Figs. 2, 3, 4). No statistically significant variations were observed between EDDY and Ultra-X in the coronal (*p* = 0.192 ve *p* = 0.108), middle (*p* = 0.595 and *p* = 0.024) and apical thirds (*p* = 0.011 and *p* > 0.999) regions.

In samples where Bioserra root canal sealer was used, Ultra-X was found to be statistically superior to CNI in the coronal region (*p* = 0.006). Additionally, Ultra-X was significantly superior to both EDDY and CNI in the middle and apical regions (*p* = 0.003, *p* < 0.001; *p* = 0.003 and *p* < 0.001, Figs. 5, 6, 7). No statistically significant variations were observed between the other groups (*p* > 0.05).

## Discussion

With the development of technology, the devices, methods and treatment approaches used in endodontic treatments have changed. With the increasing interest in endodontics, the number of endodontic treatments and the number of retreatment are gradually increasing. Due to their superior bonding and sealing properties, as well as biocompatibility bioceramic-based materials are widely utilized in root canal therapy. Consequently, we posited that the removal of bioceramic-based sealers from the root canal system would likewise provide challenges, notwithstanding the little evidence regarding their retreatability [13]. Irrigation activation techniques have been reported to be more effective in eliminating debris and smear layer from root canal irregularities than traditional syringe irrigation [21]. Following irrigation activation methods, the two distrinct bioceramic based sealers utilized in this investigation were unable to be fully removed from the root canal walls. In the present study, the CNI group displayed the highest quantity of residual filling material in all sections of the root canal wall across all groups. Both Ultra X and EDDY demonstrated statistically significantly cleaner canal walls in the coronal, middle and apical thirds compared to the CNI group, regardless of whether MTA Fillapex or Bioserra root canal sealer was used both (*p* < 0.05). The null hypothesis, which stated that there was no significant variation in canal cleanliness during the retreatment of canals filled with two different bioceramic sealer using additional irrigation activation techniques, was therefore rejected.

According to reports in the literature, resin-based and calcium silicate-based root canal sealers have comparable capacities in removing material from the root canal system [22]. Agrafioti et al. [11] stated that root canal sealers based on calcium silicate were stronger and more resistant to retreatment procedures. They also noted that the average time required to remove MTA Fillapex, Endosequence BC, and Total Fill BC sealers using a rotary file and chloroform was approximately twice that of AH Plus. Ballal et al., [18] observed lower residual concentrations of MTA Fillapex after retreatment compared to BioRoot RCS, attributing this to differences in sealer solubility. In another study, Donnermeyer et al. [19] examined the removability of BioRootRCS and MTA Fillapex using various root canal instruments. They found that the dentin walls in the BioRoot RCS group were cleaner than those of the MTA Fillapex group, attributing this difference to variations in the experimental design.

It was recommended to get support from irrigation activation devices to enhance the efficacy of root canal filling removal during retreatment protocols [12]. In one study, Ballal et al. [23] stated that ultrasonic activation of maleic acid or Dual Rinse HEDP solutions removed a greater amount of MTA Fillapex from the artifically created groves in the apical region of the root canal. Similarly, More et al. [24] found that passive ultrasonic activation with a solvent (Endosolv R) was more effective than using the solvent alone in retreating root canals filled with MTA Fillapex. In the present study, EDDY demonstrated superior performance compared to other methods in the MTAFillapex groups, while UltraX showed superior results in the BioSerra groups. These findings may be attributed to differences in the physical and chemical qualities of the sealers and the removal efficiency of the activation devices. We can attribute this to the fact that bioceramic-based sealers and the smear layer or debris have different chemical compositions. As a result, their ability to be removed following exposure to irrigation solutions activated by various methods may vary.

The potential efficiency of high-frequency (6000 Hz) sonic irrigation activation for root canal cleaning has been demonstrated in previous studies [25]. One study stated that calcium silicate-based bioceramic sealer (EndoSeal MTA) was more effectively removed using EDDY activation compared to than PUI and CNI methods in artificial grooves prepared in the apical part of the root canal [26]. Similarly, in the present study, during the retreatment of canals filled with MTAFillapex, EDDY activation left significantly less filling material in the coronal, middle and apical regions compared to the CNI and Ultra X methods. The superior performance of EDDY in the apical area may be attributed to the greater flexibility of its tip compared to the tips used in Ultra X [19]. The current findings were supported by another study, which demonstrated that EDDY significantly increased the removal of root filling material during the retreatment of distal canals in mandibular molars [27]. The superior efficiency of EDDY compared to CNI in removing residual filling material may be attributed to its acoustic conduction and cavitation effects, which directly impact the residual filling material [28].

When teeth obturated using root canal sealers based on calcium silicate, Nguyen et al. [29] found that PUI eliminated more residual filling material than CNI, except in the apical region. In contrast, the present study found that Ultra X was the most effective activation system in the Bioserra group. However contrary to these findings, Bueno et al. [30] reported that PUI did not increase the amount of residual root canal filling material removed during retreatment. Similarly, another study using micro-CT analysis found that PUI did not provide additional effectiveness in removing residual material during the retreatment process [31]. In the current study, no significant difference was observed between UltraX and CNI in removing MTA Fillapex sealer, whereas a significant difference was observed between other activation techniques in removing Bioserra sealer. In previous studies, the ultrasonic device (Obtura, Spartan Endodontics, Algonquin, IL) operated at 30% power with a frequency of 40 kHz, while the device used in the present study (Eighteeth, Ultra X, Changzhou, China) operated at a higher power setting and a frequency of 45 kHz. These discrepancies in results may be attributed to differences in the physical qualities of the sealers and the varying frequency and power settings of the ultrasonic devices used in different studies.

In this study, the coronal third of the canal was found to be the cleanest region, with lower average SEM scores in samples filled with MTA Fillapex, regardless of the experimental group. Similarly, in samples filled with Bioserra, the middle third was the cleanest region. Across all experimental groups, the apical third of the root canal system exhibited the lowest level of cleanliness. This finding was consistent with many studies in the literature [15, 32]. However, Oltra et al. [33] reported that the coronal third retained the most residual filling material. In contrast, our study found that the coronal third was better cleaned in canals filled with MTA Fillapex sealer, whereas in canals filled with Bioserra sealer, the middle third was cleaner than both the apical and coronal regions. This observation may result from the interaction between the irrigant and gas bubbles in the apical area, which could hinder the cavitation effects of the irrigant [34]. The differences between studies can be explained by factors such as variations in retreatment techniques and physical properties of the sealers used.

To ensure uniformity, this investigation focused on teeth with only one root and one canal. Therefore, the results may not be applicable to curved or multi-rooted canals. Future research should explore the removal of bioceramic-based root canal sealers from these more complex anatomies. Additionally, further in vitro and randomized controlled clinical researches are needed to investigate the effectiveness of final irrigation activation systems during retreatment on teeth with diverse canal anatomies, various root canal sealers, obturation methods, and different combinations of irrigation activation techniques. Another limitation of this investigation was the use of SEM analysis to detect residual filling material, as it could incorporate Energy Dispersive Spectrometry (EDS) for a more detailed microchemical analysis of the residual material or use micro-CT analysis to obtain more precise three-dimensional quantitative data.

Within the scope of this research, EDDY and Ultra-X irrigation activation methods provided less remaining root canal filling materials compared to CNI. We can conclude that clinicians may prefer EDDY as an irrigation activation method in the retreatment of root canals filled with MTA Fillapex root canal sealer, while they may prefer UltraX as the irrigation activation method in the retreatment of root canals filled with Bioserra root canal sealer.

## Data Availability

The data sets used and analyzed during the current study are available from the corresponding author on reasonable request.
